# DNA and RNA-based next-generation sequencing for companion diagnostic rearrangement detection in solid tumors

**DOI:** 10.1093/oncolo/oyag001

**Published:** 2026-01-09

**Authors:** Rachel B Keller-Evans, Jessica K Lee, Justin M Allen, Lei Zhong, Ole Gjoerup, Jeffrey S Ross, Amaya Gascó, Richard S P Huang

**Affiliations:** Foundation Medicine, Inc., Boston, MA 02210, United States; Foundation Medicine, Inc., Boston, MA 02210, United States; Foundation Medicine, Inc., Boston, MA 02210, United States; Foundation Medicine, Inc., Boston, MA 02210, United States; Foundation Medicine, Inc., Boston, MA 02210, United States; Foundation Medicine, Inc., Boston, MA 02210, United States; Pathology, Upstate Medical University, Syracuse, NY 13210, United States; Foundation Medicine, Inc., Boston, MA 02210, United States; Foundation Medicine, Inc., Boston, MA 02210, United States

**Keywords:** gene fusion, gene rearrangement, RNA-NGS, DNA-NGS, companion diagnostic

## Abstract

**Background:**

While a well-designed next-generation sequencing-based DNA (DNA-NGS) comprehensive genomic profiling assay can be robust for detecting genomic rearrangements (RE), concurrent RNA-based NGS (RNA-NGS) may improve overall sensitivity.

**Patients & Methods:**

We examined detection rates of tissue sequencing-based companion diagnostic (CDx) RE (*ALK*, *BRAF*, *FGFR2/3*, *MET*Ex14, *NTRK1/2/3*, *NRG1*, *RET*, and *ROS1*) in a retrospective cohort of 5129 patients who received DNA- and RNA-NGS in parallel in order to quantify the added value of concurrent DNA- and RNA-NGS over DNA-NGS alone.

**Results:**

The prevalence of CDx gene RE was 3.3% (*N* = 171) across solid tumors and 2.0% (*N* = 101) within approved tumor types (ITT) across both DNA and RNA. 20% of CDx RE ITT and 26% of CDx gene RE across solid tumors were detected with RNA-NGS only. Detection of *NRG1* and *NTRK* fusions was most improved due to the challenges of baiting these genes on DNA-NGS with 67% of *NRG1* RE+ non-small cell lung cancer (NSCLC) and 67% of *NTRK* RE+ solid tumors identified on RNA alone. A small proportion (4% ITT and 5% across all solid tumors) of CDx gene RE were detected in DNA alone in samples in which RNA could not be sequenced.

**Conclusion:**

A higher rate of CDx RE detection—most significantly for *NRG1* and *NTRK* fusions—was observed using concurrent DNA-NGS and RNA-NGS compared to DNA-NGS alone. Our results highlight the complementary nature of these methods. Given the substantial clinical benefit of RE-targeted therapies, an integrated DNA/RNA profiling strategy should be part of routine clinical care.

Implications for Practice DNA next-generation sequencing (NGS) is a reliable mainstay for detection of genomic rearrangements (RE). However, there are technical limitations to baiting and detection of RE in certain clinically important genes, which can be overcome by profiling directly expressed RE in RNA. Our findings support concurrent DNA- and RNA-NGS to enhance detection of therapeutically actionable RE across solid tumors, especially in difficult-to-bait genes in DNA-based assays, including *NRG1* and *NTRK,* for which RNA-NGS significantly improves detection.

## Introduction

Gene fusions resulting from structural genomic rearrangements (RE) are established oncogenic drivers across solid tumors.^1^ Therapeutic targeting of RE, specifically oncogenic tyrosine kinase fusions, has proven remarkably effective, with superior clinical outcomes compared to both standard of care (SOC) and matched targeted therapies (mTT) based on other genomic alteration types, eg, mutations.^2^^,^^3^ The proven clinical efficacy of this treatment approach is demonstrated by numerous U.S. Food and Drug Administration (FDA) drug approvals for agents targeting tyrosine kinase RE in various tumor types.^4–25^ Corresponding tissue sequencing-based companion diagnostic (CDx) testing for *ALK*, *BRAF*, *FGFR2/3*, *MET*, *NTRK1/2/3*, *RET*, and *ROS1* RE informing clinical use of these agents is also FDA-approved and additional CDx (eg, *NRG1*) are in development (Table 1).

**Table 1.** Current genomic rearrangement tissue sequencing-based companion diagnostic (CDx) trials supporting the initial CDx-related approval/accelerated approval indication are summarized.

CDx gene	CDx drug	CDx assay	CDx ITT	CDx indication (date of initial drug approval)	Clinical trial (phase I/II/III)	*N*	ORR (95% CI)	PFS, months (95% CI)	OS, months (95% CI)	**References**
***ALK* fusions/RE**	Crizotinib	F1CDx	NSCLC	ALK+ Adv/Met (August 26, 2011)	PROFILE 1005 [NCT00932451] (II)	*N* = 908 Central ALK testing	54% (51-57)^d^ Central ALK testing	8.4 (7.1-9.7)^d^ Central ALK testing	21.8 (19.4-24.0)^d^ Central ALK testing	Blackhall et al. (2017)^6^
	Ceritinib	F1CDx	NSCLC	ALK+ met following Crizotinib (April 29, 2014)	ASCEND-1 [NCT01283516] (I); ASCEND-3 [NCT01685138] (II)	ASCEND-1 *N *= 83 ALKi Naïve *N* = 163 Prior ALKi Tx ASCEND-3 *N* = 124 ALKi Naïve	ASCEND-1 72% (61-82)^d^ ALKi Naïve 56% (49-64)^d^ Prior ALKi Tx ASCEND-3 64% (55-72)^c^ ALKi Naïve	ASCEND-1 18.4 (11.1-NE)^d^ ALKi Naïve 6.9 (5.6-8.7)^d^ Prior ALKi Tx ASCEND-3 19.4 (10.8-29.3)^c^ ALKi Naïve	ASCEND-1 NE (19.6-NE)^d^ ALKi Naïve 16.7 (14.8-NE)^d^ Prior ALKi Tx ASCEND-3 51.3 (42.7-55.3) ALKi Naïve^d^	Kim et al. (2016)^7^; Nishio et al. (2020)^8^
	Alectinib	F1CDx	NSCLC	ALK+ Adv/Met following Crizotinib (December 11, 2015)	NCT01871805 (I/II); NCT01801111 (I/II)	*N* = 189 (PFS) *N* = 225 (OS) Pooled Analysis	51% (44-59)^c^	8.3 (7.0-11.3)^c^	29.1 (21.3-39.0)^d^	Yang et al. (2017)^4^ ; Ou et al. (2020)^5^
	Brigatinib	F1CDx	NSCLC	ALK+ following Crizotinib (April 28, 2017)	ALTA [NCT02094573] (II)	*N* = 112 90 mg Dose *N* = 11 90-180mg Dose	52% (42-61)^c^ 90 mg Dose 56% (47-66)^c^ 90-180 mg Dose	9.9 (7.4-12.8)^c^ 90 mg Dose 16.7 (11.6-21.4)^c^ 90-180 mg Dose	25.9 (18.2-45.8)^d^ 90 mg Dose 40.6 (32.5-NE)^d^ 90→180mg Dose	Gettinger et al. (2022)^5^
***BRAF*** **fusions**	Tovorafenib	F1CDx	LGG	BRAF+ Pediatric (≥6 mo) relapsed/refractory (April 23, 2024)	FIREFLY-1 [NCT04775485] (II)	*N* = 69 All *N* = 59 BRAF Fusion	67%^c,e^ All 69%^c,e^ BRAF Fusion	19.4 (16.9-NE)^c,e^ All	NE	Kilburn et al. (2024)^10^
***FGFR2*** **fusions/RE**	Pemigatinib	F1CDx	CCA	FGFR2+ Unresectable Adv/Met (April 17, 2020)	FIGHT-202 [NCT02924376] (II)	*N* = 107	36% (27-45)^c^	6.9 (6.2-9.6)^d^	21.1 (14.8-NE)^d^	Abou-Alfa et al. (2020)^11^
***FGFR3*** **fusions/RE^a^**	Erdafitinib	Therascreen (RT-PCR)	Bladder	FGFR3+ Adv/Met After Platinum (April 12, 2019)	NCT02365597 (II)	*N* = 99 All *N* = 25 FGFR Fusions	34% (25-44)^c^ All 16% (2-30)^c^ FGFR Fusions	5.5 (4.2-6.0)^d^	13.8 (9.8-NE)^d^	Loriot et al. (2019)^12^
***MET*Ex14**	Capmatinib	F1CDx	NSCLC	METEx14+ Met (May 6, 2020)	GEOMETRY Mono-1 [NCT02414139] (II)	*N* = 60 Tx Naïve *N* = 100 Prior Tx	68% (55-80)^c^ Tx Naïve 44% (34-54)^c^ Prior Tx	12.5 (8.3-18.0)^c^ Tx Naïve 5.5 (4.2-8.1)^c^ Prior Tx	21.4 (15.2-30.5)^c^ Tx Naïve 16.8 (11.6-23.8)^c^ Prior Tx	Wolf et al. (2020)^13^; Wolf et al. (2024)^14^
***NTRK1/2/3*** **fusions**	Larotrectinib	F1CDx; TrueSight	Solid Tumors	NTRK+ Adv/Met (November 26, 2018)	NCT02122913 (I); NCT02637687 (I/II); NCT02576431 (II)	*N* = 159 Pooled Analysis	79% (72-85)^d^	28.3 (22.1-NE)^d^	44.4 (36.5-NE)^d^	Hong et al. (2020)^15^
	Entrectinib	F1CDx	Solid Tumors	NTRK+ Adv/Met (August 15, 2019)	STARTRK-1 [NCT02097810] (I); STARTRK-2 [NCT02568267] (II); ALKA-373-001 (I); STARTRK-NG [NCT02650401] (I/II)	*N* = 150	61% (53-69)^c^	13.8 (10.1-20.0)^c^	37.1 (27.2-NE)^c^	Doebele et al. (2020)^16^; Krzakowski et al. (2022)^17^
***RET*** **fusions**	Selpercatinib	F1CDx; Oncomine Dx; TruSight	Solid Tumors; NSCLC, Thyroid; NSCLC	RET+ Adv/Met (May 8, 2020)	LIBRETTO-001 [NCT03157128] (I/II); LIBRETTO-431 [NCT04194944] (III)	LIBRETTO-001 *N* = 69 Tx Naïve *N* = 247 Prior Tx LIBRETTO-431 *N* = 159 Selpercatinib vs *N* = 102 Control	LIBRETTO-001 83% (72-91)^c^ Tx Naïve 62% (55-68)^c^ Prior Tx LIBRETTO-431 84% (77-89) Selpercatinib vs 63% (53-72)^c^ Control	LIBRETTO-001 22.0 (16.5-24.9)^c^ Tx Naïve 26.2 (19.3-35.7)^c^ Prior Tx LIBRETTO-431 24.8 (17.3-NE) Selpercatinib vs 11.2 (8.8-16.8)^c^ Control	LIBRETTO-001 NE (37.8-NE)^c^ Tx Naïve 47.6 (35.9-NE)^c^ Prior Tx LIBRETTO-431 NE	Zhou et al. (2023)^18^; Gautschi et al. (2025)^19^
	Praseltinib	Oncomine Dx	NSCLC	RET+ Met (September 4, 2020)	ARROW [NCT03037385] (I/II)	*N* = 233	64% (58-71)^c^	16.4 (11.0-24.1)^d^	NE	Subbiah et al. (2022)^20^; Griesinger et al. (2022)^21^
***ROS1*** **fusions**	Crizotinib	Oncomine Dx	NSCLC	ROS1+ Adv/Met (March 11, 2016)	PROFILE 1001 [NCT00585195] (I)	*N* = 53	72% (58-83)^d^	19.3 (15.2-39.1)^d^	51.4 (29.3-NE)^d^	Shaw et al. (2019)^22^
	Entrectinib	F1CDx	NSCLC	ROS1+ Met (August 15, 2019)	STARTRK-1 [NCT02097810] (I); STARTRK-2 [NCT02568267] (II); ALKA-372-001 (I)	*N* = 168	68% (60-75)^c^ TKI Naïve	15.7 (12.0-21.1)^c^	47.8 (44.1-NE)^d^	Drilon et al. (2020)^23^; Drilon et al. (2022)^24^
***NRG1*** **fusions**	Zenocutuzumab	F1RNA^b^	NSCLC, PDAC	NRG1+ Adv/Met After Prior Tx (Dec 4, 2024)	eNRGy [NCT02912949] (II)	*N* = 93 NSCLC *N* = 36 PDAC	31% (22-41)^d^ NSCLC 44% (28-62)^d^ PDAC	6.8 (5.3-7.5)^d^ NSCLC 9.2 (5.5-11.5)^d^ PDAC	NE	Schram et al. (2025)^25^

a CDx specifically for *FGFR3*::*TACC3* fusions but expanded to oncogenic fusions/RE for this analysis to match *FGFR2* CDx given similar biology.

b CDx in development.

c Independent Review Committee (IRC) assessed.

d Investigator assessed.

e RANO-HGG (Response Assessment in Neuro-Oncology for High Grade Gliomas) criteria used for assessment. Abbreviations: Adv, advanced disease; CCA, cholangiocarcinoma; F1CDx, FoundationOne CDx; F1RNA, FoundationOne RNA; ITT, in tumor type; LGG, low-grade glioma; Met, metastatic disease; NE, not evaluable; NGS, next-generation sequencing; NSCLC, non-small cell lung cancer; ORR, objective response rate; OS, overall survival; PDAC, pancreatic ductal adenocarcinoma; PFS, progression-free survival; RE, rearrangement.

DNA-based next-generation sequencing (DNA-NGS) is widely used in the clinical setting to identify genomic RE. Deliberate design features to support RE detection—including baiting within hotspot intronic regions of both target and common partner genes—enable robust detection of clinically actionable variants.^26^ However, technical limitations remain. It is difficult to efficiently bait several clinically important genes (eg, *NRG1*, *NTRK3*) due to large repetitive intronic regions^27^ and, generally, it can be difficult to resolve complex genomic RE when only sequencing DNA.^28^ RNA-based NGS (RNA-NGS) may overcome these limitations by directly detecting expressed RE in which intronic regions have been spliced out. However, the generally lower stability and assay-ready quality of RNA vs DNA,^29^ especially from archival formalin-fixed paraffin-embedded (FFPE) tissue,^30^ can result in a lower rate of successful RNA sequencing. Thus, DNA remains the cornerstone analyte for detection of actionable RE in clinical practice.

Although kinase fusions are relatively uncommon events in solid tumors, certain cancer types are enriched for these targetable alterations including non-small cell lung cancer (NSCLC), thyroid cancer, central nervous system (CNS) tumors, bladder cancer, and others.^31^ The clinical validity of RNA-NGS for detection of RE in individual cancer types, eg, NSCLC,^32,33^ has been previously reported. In this study, we evaluated detection of CDx gene RE across solid tumors using DNA-NGS and RNA-NGS performed concurrently on material co-extracted from FFPE tumor samples and quantified the added value of RNA-NGS in addition to an FDA-approved DNA-NGS assay for identifying these clinically important targets.

## Methods

### Genomic cohort selection and next-generation sequencing

We queried an institutional database of solid tumor tissue-based DNA-NGS and RNA-NGS performed during the course of routine clinical care between June and December 2024. DNA and RNA co-extracted from FFPE tumor samples were profiled, respectively, using FoundationOne^®^CDx (F1CDx^®^; DNA-NGS), an FDA-approved comprehensive genomic profiling (CGP) test that interrogates genomic alterations in 324 cancer-associated genes, including all coding exons from 309 genes, one promoter region, one non-coding RNA, and select intronic regions from 34 commonly rearranged genes,^34^^,^^35^ and FoundationOne^®^RNA (F1RNA^®^; RNA-NGS), a laboratory developed test (LDT) for the detection of RE targeting 318 genes.^36^ DNA-NGS results were analyzed for mutations (single base substitutions and short insertions/deletions), copy number changes (amplifications and homozygous deletions), and large genomic RE, as well as complex biomarkers including microsatellite instability (MSI),^37^ tumor mutational burden (TMB),^38^^,^^39^ and homologous recombination deficiency signature (HRDsig,^40^ offered as a laboratory professional service). Genomic ancestry was predicted based on analysis of single nucleotide polymorphisms (SNPs) trained on data from the 1000 Genomes Project (RRID: SCR_006828) to classify patients as belonging to one of the following subpopulations: African, East Asian, European, South Asian, and Admixed American.^41^ Clinical features (eg, age at biopsy collection, sex, biopsy site, cancer diagnosis) were extracted from test requisition forms and pathology reports. Testing was performed in a Clinical Laboratory Improvement Amendments (CLIA)-certified, College of American Pathologists (CAP)-accredited, New York State-approved laboratory (Foundation Medicine, Inc., Cambridge, MA, USA). Approval for this study, including a waiver of informed consent and a HIPAA waiver of authorization, was obtained from the WCG Institutional Review Board (Protocol No. 20152817).

### DNA and RNA RE classification

Assessed RE genes included all genes with solid tumor tissue sequencing-based CDx associations (Table 1). DNA-NGS and RNA-NGS RE analyses were run separately and manually reviewed in accordance with Foundation Medicine clinical workflows. For this analysis, DNA and RNA RE calls were considered independently for pathogenicity based on common classification rules and then compared between analytes at the gene level. Retention of the tyrosine kinase domain (TKD), or the epidermal growth factor-like domain for *NRG1*, was required (Table S1). Only predicted oncogenic fusions were considered for *BRAF*, *NRG1*, *NTRK1/2/3*, *RET*, and *ROS1*. *ALK* and *FGFR2* predicted oncogenic fusions and RE were considered consistent with broader CDx definitions. Classification of *FGFR3* RE, despite a more restricted CDx definition (*FGFR3*::*TACC3* fusions only), were extended to match the rules for *FGFR2* based on similar biology. MET exon 14 skipping (*MET*Ex14) RNA splice variants were also included; *MET*Ex14 was called in DNA as mutations affecting exon 14 splice sites.^42^ If multiple RE calls were made for the same gene in the same sample in either DNA or RNA, the event with the highest number of supporting read pairs was considered the dominant RE event in that analyte.

### Statistical analysis—DNA- and RNA-NGS genomic cohort

Statistical testing was performed using R (Version 4.2.1; RRID: SCR_001905). Fisher’s exact tests and Chi-squared tests were used, as appropriate, to assess differences in clinicogenomic features between cohorts and multiplicity corrections were made using the Benjamini-Hochberg method. All statistical tests were two-sided and performed using *P* < .05 as the threshold for statistical significance. Ninety-five percent confidence intervals for prevalence estimates were calculated in Python (Version 3.9.12; RRID: SCR_008394) using the Wilson Continuity Corrected Method (“wilsoncc”) from the statistical functions module of SciPy (Version 1.7.3; RRID: SCR_008058).

## Results

### CDx gene RE detection in solid tumors utilizing concurrent DNA-NGS and RNA-NGS

Of 5129 completed clinical orders, 90.0% (*N* = 4615) yielded reportable DNA-NGS and RNA-NGS results, while 9.9% (*N* = 508) yielded DNA-only results and 0.1% (*N* = 6) yielded RNA-only results (Figure S1A, See online supplementary material for a color version of this figure). 86.2% (438/508) of unsuccessful RNA-NGS was attributed to upstream wet lab failures (eg, insufficient RNA extraction yield or low-quality RNA precluding library construction or sequencing). One quarter (*N* = 1305) of the DNA-NGS + RNA-NGS clinical cohort had a diagnosis of non-small cell lung cancer (NSCLC), followed by colorectal (12%, *N* = 619), prostate (10%, *N* = 491), breast (8%, *N* = 389), and pancreatic (7%, *N* = 335) cancers (Figure S1B, See online supplementary material for a color version of this figure).

**Figure 1.**
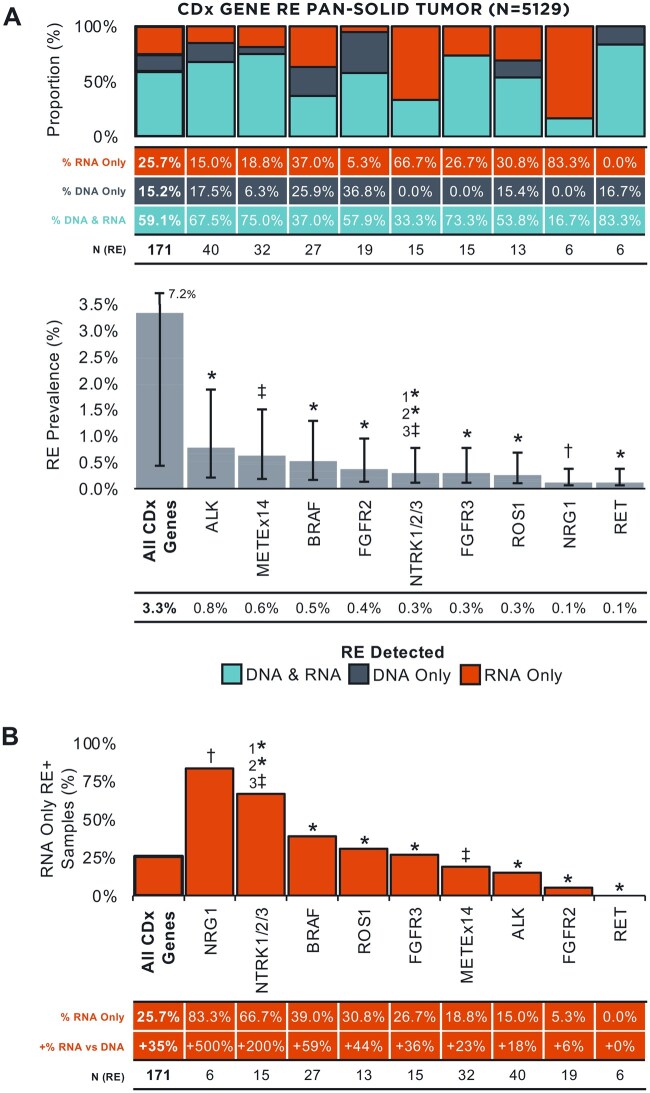
CDx gene rearrangement detection in solid tumors using concurrent DNA-/RNA-NGS (A) Prevalence of CDx gene RE detected across solid tumors (*N* = 5129) by gene (*Bottom Panel*) and proportion detected in DNA vs RNA (*Top Panel*). 95% CI are indicated. (B) The additional value of RNA for CDx gene RE detection across solid tumors. Asterisks (*) denote genes with complete exonic and select hotspot intronic coverage on F1CDx. Double daggers (‡) denote complete exonic but no intronic coverage on F1CDx. Daggers (†) denote genes with no exonic or intronic coverage on F1CDx. However, there is select hotspot intronic coverage of common partner genes for target genes lacking intronic coverage (‡ and †, eg, *CD74* and *SDC4* for *NRG1* and *ETV6* for *NTRK3*). *N* = 2 samples harbored multiple CDx gene RE (NSCLC w/*ALK* [DNA & RNA] + *BRAF* [RNA Only]; CCA w/*FGFR2* [DNA & RNA] + *BRAF* [RNA Only]); for Figure 1A-B, “All CDx Genes” only includes the more clinically relevant (ITT) of the two RE events (ie, *ALK* for NSCLC and *FGFR2* for CCA). CDx, companion diagnostic; CNS, central nervous system; NSCLC, non-small cell lung cancer; RE, rearrangement.

A total of 173 oncogenic CDx gene RE detected in 171 samples were identified across all solid tumors (*N* = 5129; Figure S2, See online supplementary material for a color version of this figure; Table S2) using DNA-NGS and/or RNA-NGS: 59.1% were detected on both DNA and RNA, 15.2% were detected on DNA only, and 25.7% were detected on RNA only (Figure 1A). *ALK* (0.8% prevalence, 40/5129) and *MET* (0.6%, 32/5129) were the most commonly altered CDx genes across all solid tumors, largely driven by the high volume of NSCLC in the cohort, while *NRG1* (0.1%, 6/5129) and *RET* (0.1%, 6/5129) were the least commonly altered. Detection of RE by DNA vs RNA varied widely across CDx genes. In the two most commonly altered CDx genes, 15.0% (6/40) of *ALK* RE were detected in RNA only (+18% detection over DNA alone [6 RNA-only detected RE:34 DNA-detected RE]) and 18.8% (6/32) of *MET*Ex14 alterations were detected in RNA only (+23% [6:26]). The CDx genes with the most added value from RNA-NGS were *NRG1* (83.3% [5/6] detected in RNA only; +500% [5:1]) and *NTRK* (66.7% [10/15]; +200% [10:5]), while *FGFR2* (5.3% [1/19]; +6% [1:18]) and *RET* (0.0% [0/6]) showed modest or no benefit from the addition of RNA (Figure 1B). Because F1CDx baiting for individual *NTRK* genes varies, we provide a breakdown of detection of *NTRK* fusions (Figure S3A, See online supplementary material for a color version of this figure) and the added value of RNA (Figure S3B, See online supplementary material for a color version of this figure) for individual *NTRK* genes (*NTRK1*, *NTRK2*, *NTRK3*) across solid tumors. At the cohort level, the addition of RNA increased the prevalence of detected CDx gene RE across all solid tumors from 2.5% (127/5129) to 3.3% (171/5129).

**Figure 2.**
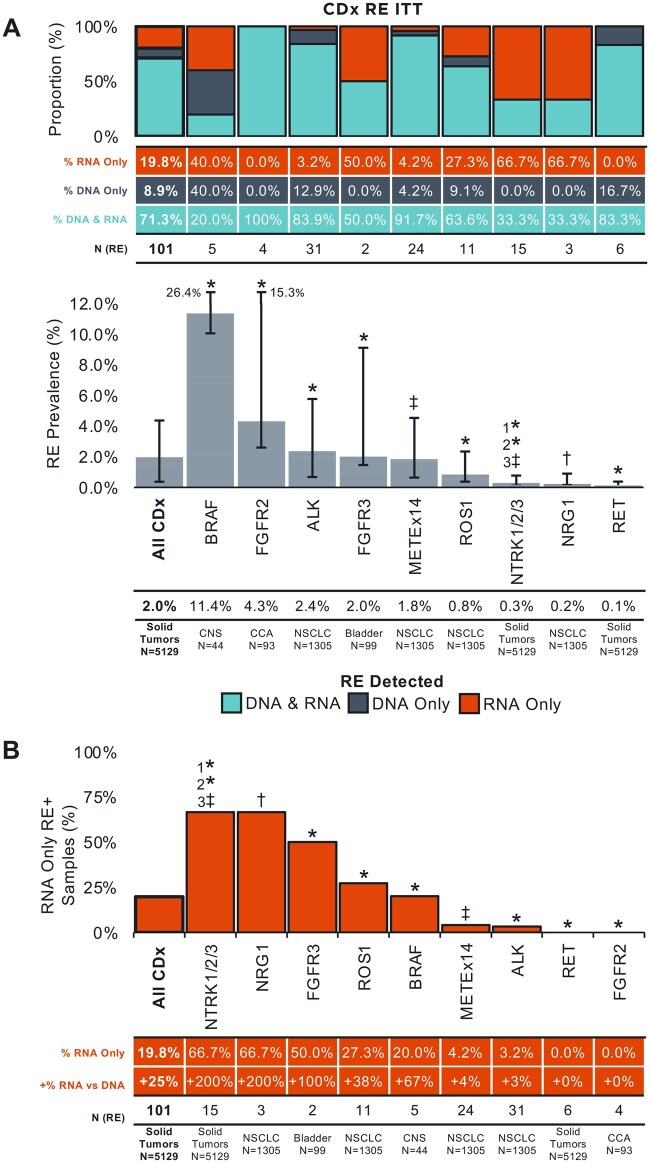
CDx rearrangement detection in approved tumor types (ITT) using concurrent DNA-/RNA-NGS (A) Prevalence of CDx RE detected ITT by gene (*Bottom Panel*) and proportion detected in DNA vs RNA (*Top Panel*). 95% CI are indicated. (B) The additional value of RNA for CDx RE detection ITT. Asterisks (*) denote genes with complete exonic and select hotspot intronic coverage on F1CDx. Double daggers (‡) denote complete exonic but no intronic coverage on F1CDx. Daggers (†) denote genes with no exonic or intronic coverage on F1CDx. However, there is select hotspot intronic coverage of common partner genes for target genes lacking intronic coverage (‡ and †, eg, *CD74* and *SDC4* for *NRG1* and *ETV6* for *NTRK3*). CCA, cholangiocarcinoma; CDx, companion diagnostic; CNS, central nervous system; ITT, in tumor type; NSCLC, non-small cell lung cancer; RE, rearrangement.

Next, we analyzed the subset of RE detected in the approved tumor type (ITT) for the relevant CDx: *ALK*, *MET* Ex14, and *ROS1* in NSCLC; *NRG1* in NSCLC and pancreatic ductal adenocarcinoma (PDAC); *BRAF* in CNS tumors; *FGFR2* in cholangiocarcinoma (CCA); *FGFR3* in bladder cancer; and *NTRK* and *RET* in all solid tumors. A total of 101 predicted oncogenic CDx RE-positive samples were identified ITT with DNA-NGS and/or RNA-NGS (Figure S2, See online supplementary material for a color version of this figure; Table S3): 71.3% were detected on both DNA and RNA, 8.9% were detected on DNA only, and 19.8% were detected on RNA only (Figure 2A). *BRAF* (11.4% in CNS, 5/44) and *FGFR2* (4.3% in CCA, 4/93) were detected at the highest prevalence ITT, while *NRG1* (0.2% in NSCLC, 3/1305) and *RET* (0.1% across solid tumors, 6/5129) were the least commonly altered. The CDx genes with the most added value from RNA-NGS were *NTRK* (66.7% [10/15] detected in RNA only; +200% detection over DNA alone [10 RNA-only detected RE:5 DNA-detected RE]) and *NRG1* (66.7% [2/3]; +200% [2:1]), while *RET* (0.0% [0/6]) and *FGFR2* (0.0% [0/4]) demonstrated no benefit from the addition of RNA (Figure 2B). Note that no *NRG1* fusions were detected across 126 PDAC profiled in our cohort. At the cohort level, the addition of RNA increased the prevalence of CDx RE detected ITT from 1.6% to 2.0%.

### Clinical characteristics of CDx gene RE-positive vs negative solid tumors

In the pan-tumor cohort, the clinical characteristics of CDx gene RE-positive vs negative patients were similar except for genomic ancestry (*P* = .004), likely due to patients with Asian ancestry constituting a higher proportion of patients who harbored CDx RE (East Asian: 7.6% [13/171] CDx Gene RE+ vs 3.3% [162/4958] CDx Gene RE-; South Asian: 2.9% [5/171] CDx Gene RE+ vs 1.0% [50/4958] CDx Gene RE-), and TMB, with a lower percentage of CDx gene RE+ patients having elevated TMB ≥10 mut/Mb (7.0% [12/171] vs 14.9% [737/4958] of CDx Gene RE- patients; *P* = .028) (Table 2). Clinical characteristics of CDx gene RE-positive vs negative patients with NSCLC also showed some differences (Table S4). While the median age at biopsy was comparable at 69 years, a higher proportion of CDx gene RE+ patients had young onset (<50 years) disease (12.8% vs 3.3% with CDx Gene RE- NSCLC; *P* = .001). Lung adenocarcinoma (LUAD) represented a higher relative proportion of CDx gene RE+ patients (87.2% CDx Gene RE+ vs 61.5% CDx Gene RE-; *P* = .001) compared to other histologies. Similar to CDx gene RE+ patients in the overall cohort, a lower percentage had elevated TMB ≥10 mut/Mb (7.0% vs 28.5% with CDx Gene RE- NSCLC; *P* < .001). Clinical characteristics were also compared for RE-positive vs negative patients with regard to the two pan-solid tumor CDx: *NTRK1/2/3* (Table S5) and *RET* (Table S6). While comparison of CDx RE-positive vs CDx RE-negative cohorts for both genes was limited by small *N*, notable findings for *NTRK* included a skewed cancer type distribution (*P* = .005) with 33.3% of *NTRK*+ patients having a diagnosis of pancreatic cancer vs 6.5% of *NTRK*- patients and a trend towards younger age at biopsy for *NTRK*+ patients (median 59 [IQR: 29, 70] years for *NTRK*+ vs median 67 years [IQR: 60, 75] for NTRK-, with the *NTRK*+ cohort inclusive of 4 pediatric patients [≤18 years of age]).

**Table 2.** Clinicogenomic characteristics of concurrent DNA-/RNA-NGS solid tumor cohort stratified by CDx gene rearrangement status.

Characteristic	Overall	CDx gene RE (+)	CDx gene RE (−)	*P*
***N***	5129	171	4958	**—**
**Age at Bx (years)**				
**Median [IQR]**	67 [59, 75]	68 [59, 74]	67 [60, 75]	.68
**<50 years, *N* (%)**	503 (9.8)	26 (15.2)	477 (9.6)	.06
**Sex, *N* (%)**				.68
**Female**	2406 (46.9)	84 (49.1)	2322 (46.8)	—
**Male**	2723 (53.1)	87 (50.9)	2636 (53.2)	—
**Genomic ancestry** ^a^ **, *N* (%)**				.004*
**AFR**	652 (12.7)	17 (9.9)	635 (12.8)	—
**AMR**	558 (10.9)	25 (14.6)	533 (10.8)	—
**EAS**	175 (3.4)	13 (7.6)	162 (3.3)	—
**EUR**	3541 (69.0)	110 (64.3)	3431 (69.2)	—
**SAS**	55 (1.1)	≤5 (2.9)	50 (1.0)	—
**Unknown**	148 (2.9)	≤5 (0.6)	147 (3.0)	—
**Biopsy site, *N* (%)**				.68
**Primary**	2543 (49.6)	91 (53.2)	2452 (49.5)	—
**Lymph node**	486 (9.5)	19 (11.1)	467 (9.4)	—
**Metastasis**	1186 (23.1)	33 (19.3)	1153 (23.3)	—
**Unknown**	914 (17.8)	28 (16.4)	886 (17.9)	—
**MSI status**				.76
**MSI-H**	106 (2.1)	2 (1.2)	104 (2.1)	—
**Not MSI-H**	4520 (88.1)	151 (88.3)	4369 (88.1)	—
**Unknown**	503 (9.8)	18 (10.5)	485 (9.8)	—
**TMB**				.028*
**≥10 Mut/Mb**	749 (14.6)	12 (7.0)	737 (14.9)	—
**<10 Mut/Mb**	3854 (75.1)	138 (80.7)	3716 (74.9)	—
**Unknown**	526 (10.3)	21 (12.3)	505 (10.2)	—

*P*-values are FDR corrected and reflect comparison between the CDx RE(+) and (−) populations. Asterisks (*) indicate a significant *P* value (<.05).

a Genomic ancestry reflects 1000 Genomes Project superpopulations. Abbreviations: AFR, African; AMR, admixed American; CCA, cholangiocarcinoma; CDx, companion diagnostic; EAS, East Asian; EUR, European; ITT, in tumor type; MSI-H, microsatellite instability-high; NSCLC, non-small cell lung cancer; NT, not tested; PDAC, pancreatic ductal adenocarcinoma; RE, rearrangement; SAS, South Asian; TMB, tumor mutational burden.

### CDx gene RE detection in DNA vs RNA

**Figure 3.**
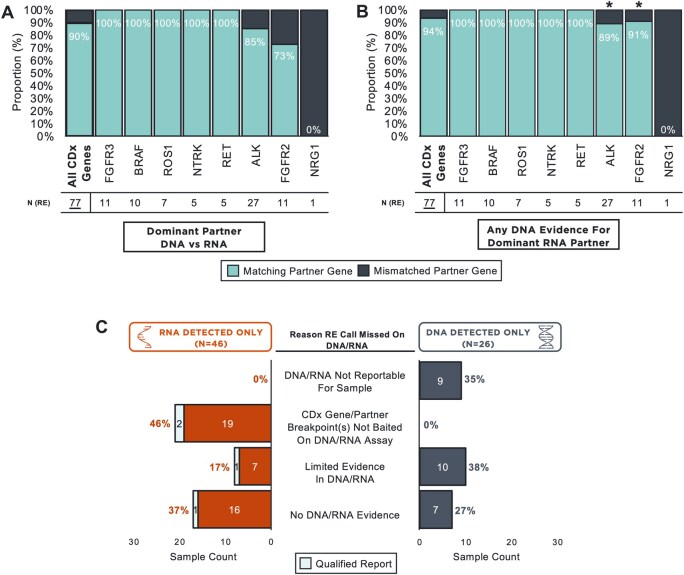
DNA/RNA concurrence for detected CDx gene rearrangements (A) Proportion of tumors with CDx gene RE detected on both DNA-NGS and RNA-NGS (*N* = 77) for which the dominant partner (ie, partner gene in RE event with the highest read support) was matched vs mismatched between RNA and DNA. (B) Proportion of tumors with CDx gene RE detected on both DNA-NGS and RNA-NGS (*N* = 77) for which there was any evidence on DNA-NGS (≥1 supporting read) for the dominant partner detected on RNA-NGS and agreement between RNA and DNA. Asterisks (*) denote genes with improved agreement using any DNA evidence to support the dominant partner in RNA. (C) Putative reasons for discordance between DNA and RNA for detected RE when detected in RNA only (*N* = 46) or in DNA only (*N* = 26). CDx, companion diagnostic; RE, rearrangement.

Canonical partners were the most common RE partners for each CDx gene across both DNA and RNA, as expected: *EML4* with *ALK*, *KIAA1549* with *BRAF*, *TACC2* with *FGFR2*, *TACC3* with *FGFR3*, *ETV6* with *NTRK3*, *KIF5B* with *RET*, and *CD74* with *ROS1* (Figure 4A**-**J, See online supplementary material for a color version of this figure). We assessed fusion partner agreement between analytes in the subset of tumors in which a predicted oncogenic CDx gene RE was detected on both DNA-NGS and RNA-NGS (*N* = 77). The dominant partner (ie, partner in the RE call with the highest level of read support) was largely the same (90% [69/77]) across all CDx gene RE events with 100% matching for *BRAF*, *FGFR3*, *NTRK*, *RET*, and *ROS1*. Matching was lower for *ALK* (85% [23/27]), *FGFR2* (73% [8/11]), and *NRG1* (0% [0/1]) (Figure 3A). If any DNA evidence for the dominant RNA-detected partner was instead considered, partner gene matching for *ALK* and *FGFR2* improved to 89% (24/27) and 91% (10/11), respectively (Figure 3B).

We assessed putative explanations for the observed discordance in CDx gene RE detection between DNA and RNA (Figure 3C). Forty-six percent (21/46) of RE detected on RNA only had no observable evidence in DNA due to baiting differences between F1RNA and F1CDx, whereas 37% (17/46) of RNA-only events were not ascribable to baiting limitations. The latter included six *MET*Ex14 events in RNA with no corresponding *MET* mutation detected in DNA and two tumors with a qualified DNA report suggesting the RE event could have been missed due to low tumor purity in these samples. Notably, the RNA read counts for some of these missed events were low (29% [5/17] with ≤25 supporting read pairs). There was limited evidence on DNA for the remaining 17% (8/46) of RNA only detected events, eg, samples with messy clips observable at breakpoint locations that were not called by the RE pipeline, RE calls filtered out by the pipeline due to QC issues, and RE considered variants of uncertain significance (VUS) by either the pipeline or pathogenicity classification in our study. Conversely, 35% (9/26) of RE events detected on DNA only were in samples for which RNA was not reportable. There was limited evidence on RNA for an additional 38% (10/26) of events, while was there was no evidence for 27% (7/26) of events, which notably included three DNA events with low-level read support (<10 read pairs), potentially indicating subclonal RE.

### Clinical outcomes for RE matched targeted therapy in advanced NSCLC

While clinical outcomes were not available for patients profiled with F1CDx + F1RNA at the time of analysis, we explored real-world outcomes for patients with advanced NSCLC with an identified CDx RE (*ALK* fusion/rearrangement, *MET*Ex14, *RET*/*ROS1* fusion) on DNA-NGS (tissue or liquid) who received 1 L mTT (*N* = 197) vs SOC (*N* = 45) in the Flatiron Health-Foundation Medicine Clinico-Genomic Database (FH-FMI CGDB; Supplementary Materials & Methods, Figure S5A, See online supplementary material for a color version of this figure). The clinical characteristics of patients varied between the cohorts, including statistically significant differences in age (median 67 years [IQR: 56, 75] mTT vs 77 [IQR: 69, 82] SOC, *P* < .001) and smoking history (41.6% mTT vs 71.1% SOC, *P* = .001) (Table S7). After balancing for covariates (Figure S5B, See online supplementary material for a color version of this figure), we observed both improved real-world progression-free survival (rwPFS; 10.4 months mTT vs 8.2 months SOC, *P* = .04; Figure S5C, See online supplementary material for a color version of this figure) and real-world overall survival (rwOS; 29.9 months mTT vs 18.3 months SOC, *P* = .02; Figure S5D, See online supplementary material for a color version of this figure) for patients receiving mTT. One- and two-year survival rates were 73.0% (95% CI, 65.9-80.8) and 57.1% (95% CI, 49.4-66.1), respectively, for mTT vs 52.8% (95% CI, 35.0-79.8) and 43.2% (95% CI, 25.9-72.2) for SOC.

## Discussion

In a retrospective analysis of patients with advanced solid tumors with parallel DNA-NGS and RNA-NGS in real-world clinical practice, we found that while DNA-NGS is robust for detecting many CDx gene RE, 20% of CDx RE+ samples ITT and 26% of CDx gene RE+ samples across all solid tumors were detected with RNA-NGS only. This equates to a 25% increase in identified CDx RE+ patients and a 35% increase in identified patients with a potentially actionable CDx gene RE pan-tumor. The benefit of concurrent testing was demonstrated across CDx RE genes with the largest improvements in detection with the addition of RNA-NGS observed for *NRG1* and *NTRK* fusions. This was expected since baiting for RE on DNA-NGS for these genes is challenging due to the presence of large repetitive intronic regions^27^; as a result, only exonic regions of *NTRK3* are baited on F1CDx, and *NRG1* is not a baited gene. However, a subset of *NTRK3* and *NRG1* fusions are detectable using F1CDx due to baiting of common partner genes (*ETV6* for *NTRK3, CD74* and *SDC4* for *NRG1*) and, indeed, 1 of 9 *NTRK3* fusions (11.1%) was concurrently detected in both DNA and RNA due to this design feature. Notably, RNA-NGS had significant added value for the detection of *NTRK* fusions which are FDA-approved tumor-agnostic biomarkers associated with striking responses to targeted TRK inhibitors, eg, 79% ORR (95% CI, 72-85) to larotrectinib in a pooled analysis of three key clinical trials (NCT02122913, NCT02637687, NCT02576431)^15^ and 61% ORR (95% CI, 53-69) to entrectinib in integrated analysis of three trials (EudraCT 2012-000148-88, NCT02097810, NCT02568267)^16^^,^^17^ which led to FDA approval of both drugs.

The impact of concurrent testing for some CDx RE was difficult to observe in this study, in part due to low *N*. There was no additional benefit of RNA-NGS for *RET* in solid tumors as 100% (6 of 6) of detected CDx fusions involved intron 11, which is baited on F1CDx. Likewise, there was no additional benefit of RNA-NGS for *FGFR2* in CCA as 100% (4 of 4) of detected CDx RE involved intron 17, which is also baited on F1CDx. Generally, the benefit of RNA-NGS was somewhat mitigated for CDx ITT compared to CDx gene RE detected across solid tumors (20% vs 25% of RE detected in RNA alone, respectively), likely because breakpoint hotspots in key cancer types relevant for each gene were considered when designing the F1CDx bait set. While we would expect to observe a degree of added benefit for all genes (including *RET* and *FGFR2*) in a larger dataset, this finding speaks to the value of thoughtful assay design such that no *RET* CDx+ or *FGFR2* CDx+ RE was undetected on DNA-NGS in the current study.

Overall, concurrent detection of a predicted oncogenic CDx gene RE with both DNA-NGS and RNA-NGS was observed in 59% of RE+ solid tumors and 71% of RE+ ITT. When CDx gene RE were detected in both DNA and RNA, the dominant partner gene generally matched (90% across all CDx gene RE and 100% matched for *BRAF*, *FGFR3*, *NTRK*, *RET*, and *ROS1*). *ALK* (85% matched) and *FGFR2* (73% matched) had less agreement due to more liberal CDx definitions, which encompass both fusions and RE, such that there were often multiple RE candidate calls in DNA (and sometimes in RNA) with varying levels of read support. When considering all DNA evidence, 94% of cases had matching partners. Thus, there should be very high confidence in CDx gene RE when detected concurrently on DNA-NGS and RNA-NGS. In exploring putative reasons for CDx gene RE events not concurrently detected by both assays, we observed that a substantial proportion (46%) of events detected on RNA missed by DNA were due to baiting differences between F1RNA and F1CDx, such as a lack of intronic baiting on DNA where breakpoints occurred (eg, an *EML4*-*ALK* fusion with breakpoint in *ALK* intron 17 when only introns 18 and 19 are baited on F1CDx). While the rate of reportable RNA-NGS was high (90% for samples with reportable DNA-NGS), a small proportion of CDx gene RE—4% (4/102) ITT and 5% (9/173) across all solid tumors—were identified in DNA alone due to unsuccessful RNA sequencing. The clinical significance of CDx gene RE events detected on RNA or DNA alone due to unclear reasons (54% of RNA only and 65% of DNA only with either limited or no evidence of RE in the other analyte) remain to be explored. A limitation of this study is the lack of clinical data and/or orthogonal testing to investigate the clinical utility of CDx gene RE events detected on RNA vs DNA. RE calling and functional interpretation are complex and our study relied on a single commercial platform for detection and a set of rules for predicting pathogenicity. RE classification may have been determined differently by different investigators, which would directly impact the findings/conclusions of the study.

Taken together, our findings emphasize the complementary nature of DNA-NGS and RNA-NGS for the detection of CDx RE in solid tumors and support a concurrent testing approach. Our findings are consistent with prior studies of integrated DNA and RNA sequencing in which RNA sequencing improved sensitivity for actionable RE detection^32^^,^^33^ with the added significance of (1) benchmarking the added value of RNA-NGS against the capabilities of an FDA-approved DNA-NGS assay with CDx approvals in 7 of the 9 CDx RE genes analyzed and (2) reflecting the real-world experience of the added value of RNA-NGS by not restricting to samples with successful sequencing of both analytes. While most CDx RE genes analyzed in this study have coverage on both F1CDx and F1RNA (excepting *NRG1* and intronic *NTRK3*), there is significant non-overlap between these assays including 193 unique RE genes with coverage on F1RNA with clinical implications for diagnosis (eg, *HMGA1/2*, *COL1A1*, *CREB3L1/2* and others in sarcomas^43^), prognosis (eg, *TFE3* in renal cell carcinoma^44^), and treatment selection (eg, *RSPO3* in colorectal cancer for WNT pathway inhibition^45^^,^^46^). Conversely, there are 199 unique cancer-associated genes with coverage on F1CDx for the detection of non-RE alteration types (mutations, amplifications, and homozygous deletions) along with detection of these other alteration classes in the 125 shared F1RNA/F1CDx genes, as well as complex biomarkers (TMB, MSI, HRDsig). Thus, concurrent DNA-/RNA-NGS enables more complete tumor profiling and genomic contexualization,^47^^,^^48^ which needs to be a consideration for precision oncology moving forward.

Pathogenic RE are relatively uncommon events in the unselected cancer population. In our cohort, CDx gene RE were seen in 3.3% of patients, with 0.9% of patients identified only by RNA-NGS. However, the clinical impact of detection of an actionable RE for an individual patient is substantial with CDx RE mTT achieving high response rates (16%-84% in key clinical trials leading to FDA drug approvals) and months of added survival benefit (5.5-28.3 months PFS, 13.8-51.4 months OS) (Table 1).^4–25^ In a study of 59 patients with various cancer types harboring actionable gene fusions who received mTT as part of a Phase I clinical trial program, while response rates were comparable to historical mTT, PFS (7.1 months [95% CI, 4.8-15.4]) and OS (19.6 months [95% CI, 10.8-29.4] were extended compared to historical rates for both mTT for mutations (PFS = 5.2 months, OS = 13.4 months) and unmatched therapy (PFS = 2.2 months, OS = 9.0 months).^2^ In another study, clinical outcomes were compared for 79 patients with gene fusion-positive solid tumors identified using Foundation Medicine tissue DNA-NGS. Again, patients receiving fusion mTT experienced prolonged PFS (11.6 months [95% CI, 4.0-35.4]) compared to those receiving both unmatched therapy (5.0 months [95% CI, 3.3-8.8]) and mTT for other alteration types (4.0 months [95% CI, 3.3, Not Estimated]).^3^ Clinical outcomes were not available for the F1CDx + F1RNA cohort at the time of analysis. However, we did perform a retrospective real-world outcomes analysis of patients with advanced NSCLC harboring CDx RE identified on F1CDx alone who received 1 L mTT vs SOC therapy and, similarly, saw marked improvements in rwPFS (10.4 vs 8.2 months, *P* = .04), rwOS (29.9 vs 18.3 months, *P* = .02), and 1- and 2-year survival rates (73.0% vs 52.8% and 57.1% vs 43.2%, respectively) with the use of RE mTT. Clinical guidelines have evolved in recognition of the demonstrated clinical importance of detecting genomic RE driver events with regard to RNA profiling. National Comprehensive Cancer Network (NCCN) guidelines for NSCLC, in addition to recommending broad, panel-based approaches when feasible, describe RNA-NGS as the preferred method for detecting *RET* fusions; state that RNA-NGS may offer improved detection of *NTRK* fusions and *MET*Ex14 skipping events; and also suggest consideration of RNA-NGS when prior testing (eg, DNA-NGS) identifies no oncogenic drivers in order to “maximize detection of fusion events”.^49^ An American Society for Clinical Oncology (ASCO) opinion considered RNA-based approaches to be generally superior to DNA-based methods for detecting expressed fusions and also suggest RNA-based testing be pursued for patients for whom an oncogenic driver was not identified with a DNA multigene panel or for whom SOC options have been exhausted.^50^ In short, every effort should be made to identify cancer patients with actionable RE drivers, supporting a concurrent DNA-/RNA-NGS approach. A DNA-NGS plus RNA-NGS strategy should also be a consideration during drug development in order to capture the largest possible population of eligible patients for clinical trials and to potentially refine relevant biomarkers by clarifying/confirming the functional significance of detected RE. Importantly, the additional value of RNA for detection of diagnostic and prognostic RE in genes not analyzed in this study is expected to further support RNA as a key analyte for profiling particular cancer types (eg, sarcomas, salivary gland, and CNS tumors), which future studies of this cohort will explore.

## Conclusions

This study adds to existing clinical evidence supporting utilization of concurrent DNA-NGS and RNA-NGS in solid tumors by demonstrating that RNA-NGS augments detection of CDx RE, most significantly in *NRG1* and *NTRK*, over DNA-NGS alone. As detection of CDx RE has significant clinical implications for patients, unlocking proven efficacious treatment options, integrated DNA-NGS and RNA-NGS should be a standard offering in the clinic.

## Supplementary Material

oyag001_Supplementary_Data

## Data Availability

The authors declare that all relevant aggregate data supporting the findings of this study are available within the article and its supplementary information files. The data that support the findings of this study originated from Foundation Medicine, Inc. In accordance with the Health Insurance Portability and Accountability Act, we do not have IRB approval or patient consent to share individualized patient genomic data, which contains potentially identifying or sensitive patient information and cannot be reported in a public data repository. Foundation Medicine is committed to collaborative data analysis and has well established and widely used mechanisms by which qualified researchers can query our core genomic database of >1 000 000 de-identified sequenced cancers. Academic researchers can submit a proposal to the Foundation Medicine Data Collaborations Committee and, if approved, the researcher/institution will be required to complete a Data Usage Agreement. More information and mechanisms for data access can be obtained by contacting the corresponding author or the Foundation Medicine Data Governance Council at data.governance.council@foundationmedicine.com.
