# Supplementary material for: DNA and RNA-based next-generation sequencing for companion diagnostic rearrangement detection in solid tumors
Source: Oncologist. 2026 Jan 9;31(3):oyag001. doi: 10.1093/oncolo/oyag001 (PMC12923117; doi:10.1093/oncolo/oyag001)
Supplement: oyag001_Supplementary_Data [file oyag001_supplementary_data.pdf]

SUPPLEMENTARY DATA

A

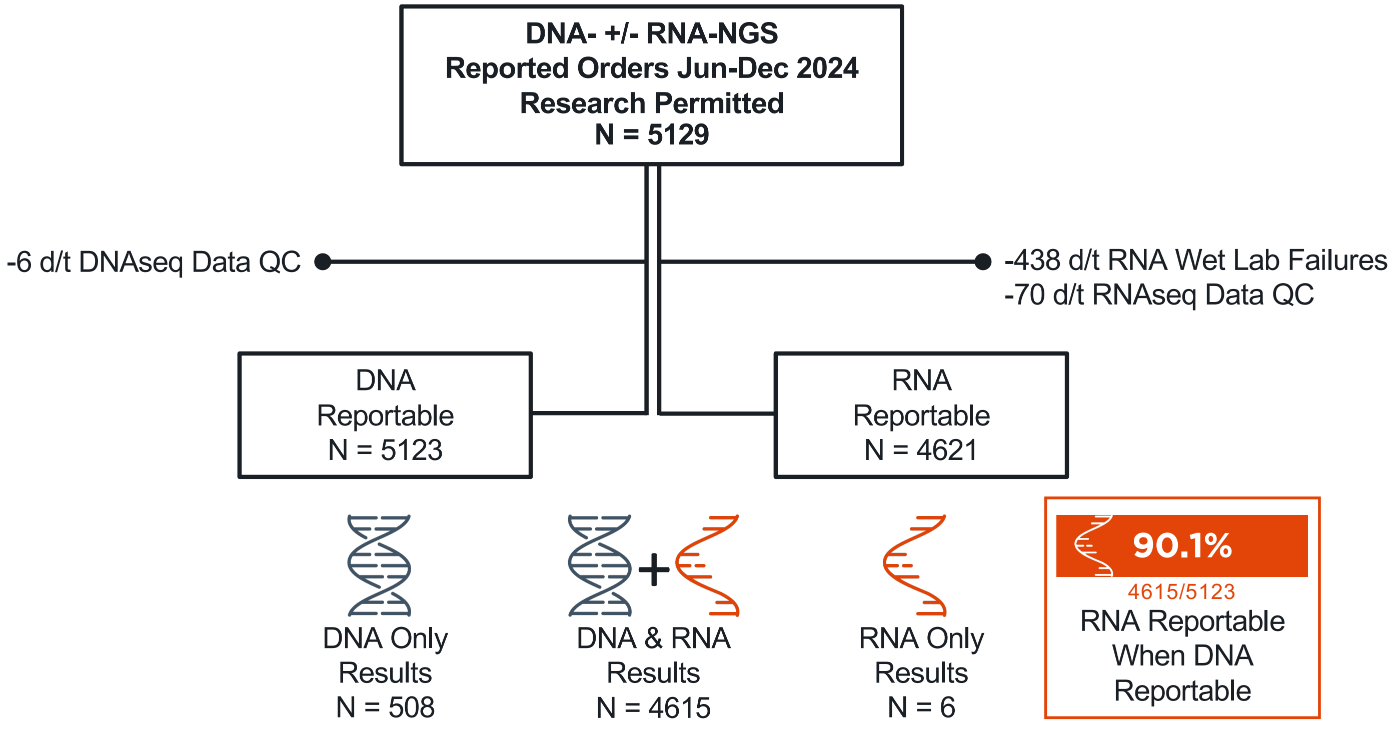

B

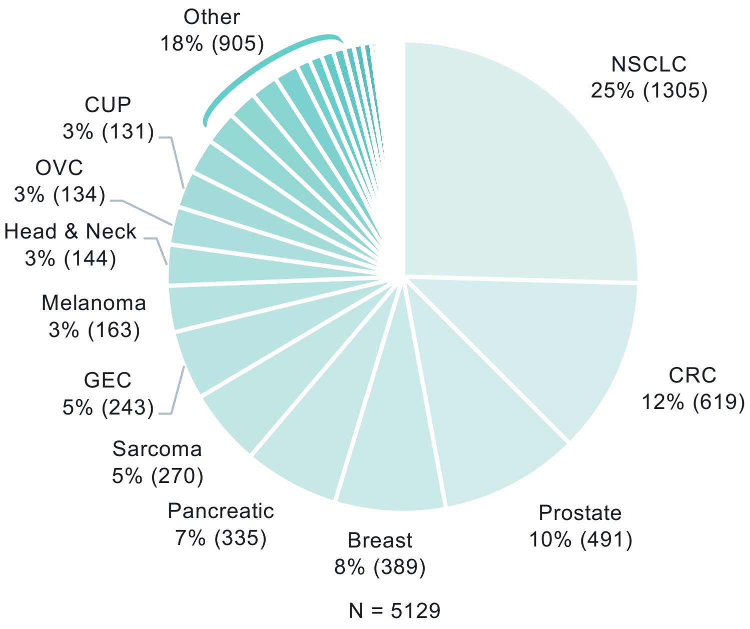

Supplementary Figure 1. Concurrent DNA-/RNA-NGS Cohort A) Reporting status of solid tumor samples sent for concurrent DNA-/RNA-NGS with reportable results. B) Cancer type breakdown of cohort. CRC, Colorectal Cancer; CUP, Unknown Primary Carcinoma; GEC, Gastroesophageal Cancer; NSCLC, Non-Small Cell Lung Cancer; OVC, Ovarian Cancer.

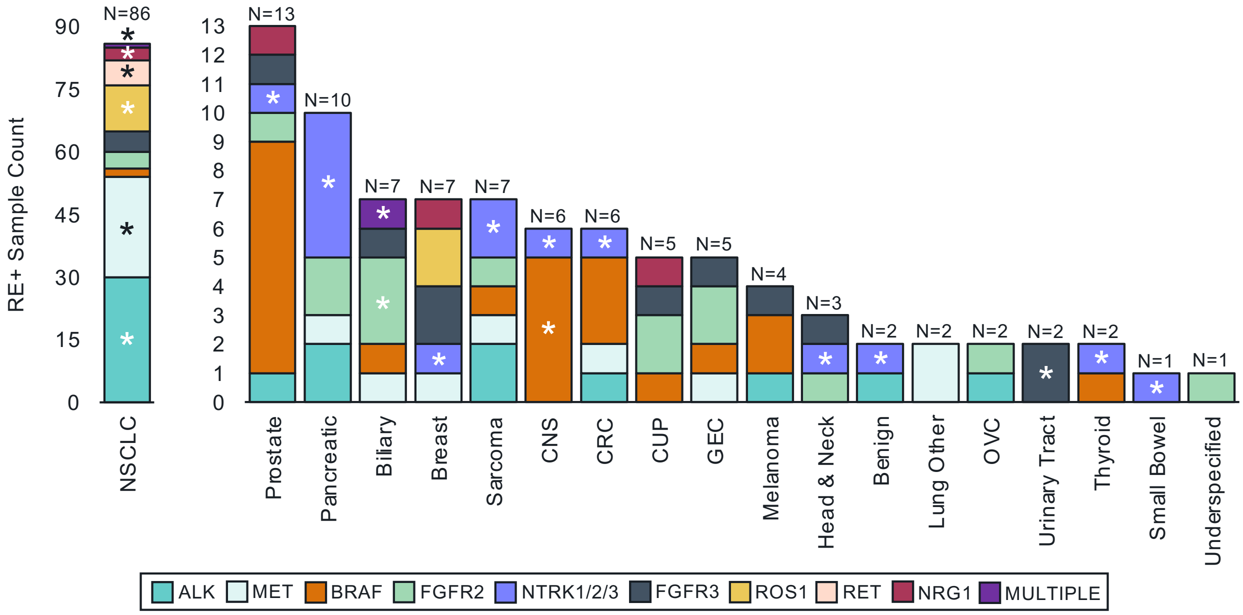

Supplementary Figure 2. CDx Gene RE+ Samples (N = 171) Detected Using Concurrent DNA-/RNA-NGS Stratified By Cancer Type Asterisks (*) denote genes that are ITT for the relevant CDx. N = 2 samples harbored multiple CDx gene RE (NSCLC w/ *ALK* [DNA & RNA] + *BRAF* [RNA Only]; CCA w/ *FGFR2* [DNA & RNA] + *BRAF* [RNA Only]). CNS, Central Nervous System; CDx, Companion Diagnostic; CRC, Colorectal Cancer; CUP, Unknown Primary Carcinoma; GEC, Gastroesophageal Cancer; NSCLC, Non-Small Cell Lung Cancer; OVC, Ovarian Cancer; RE, Rearrangement.

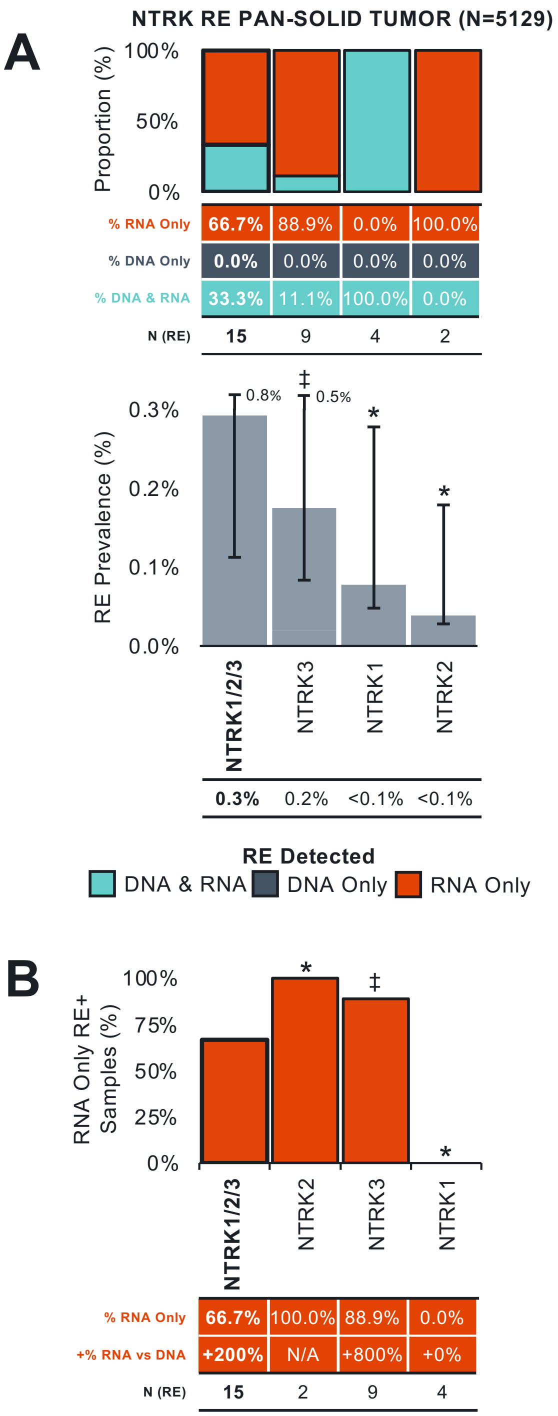

Supplementary Figure 3. NTRK Fusion Detection In Solid Tumors Using Concurrent DNA-/RNA-NGS
A) Prevalence of NTRK fusions detected across solid tumors (N = 5129) by gene (*Bottom Panel*) and proportion detected in DNA versus RNA (*Top Panel*). 95% CI are indicated. B) The additional value of RNA for NTRK fusion detection across solid tumors. **NTRK1/2* have complete exonic and select hotspot intronic coverage on F1CDx. ‡*NTRK3* has complete exonic, but no intronic coverage on F1CDx. However, there is select hotspot intronic coverage of *ETV6*, a common *NTRK3* partner gene. CDx, Companion Diagnostic; RE, Rearrangement.

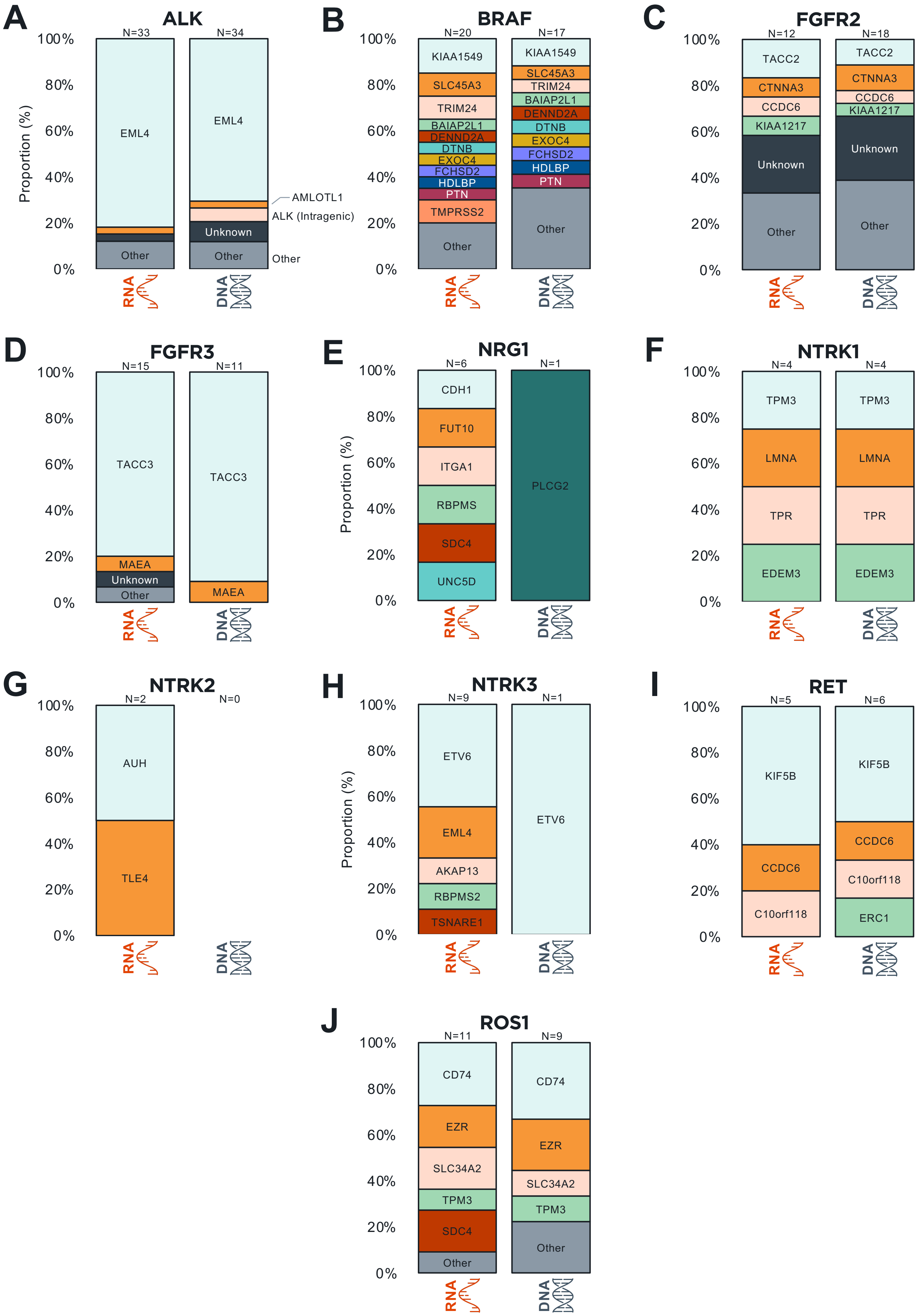
A
ALK
N=33
N=34
100%
80%
60%
40%
20%
0%
Proportion (%)
EML4
EML4
AMLOTL1
ALK (Intragenic)
Unknown
Other
Other
Other
RNA
DNA
B
BRAF
N=20
N=17
KIAA1549
SLC45A3
TRIM24
BAIAP2L1
DENND2A
DTNB
EXOC4
FCHSD2
HDLBP
PTN
TMPRSS2
Other
C
FGFR2
N=12
N=18
TACC2
CTNNA3
CCDC6
KIAA1217
Unknown
Other
D
FGFR3
N=15
N=11
TACC3
MAEA
Unknown
Other
E
NRG1
N=6
N=1
CDH1
FUT10
ITGA1
RBPMS
SDC4
UNC5D
PLCG2
F
NTRK1
N=4
N=4
TPM3
LMNA
TPR
EDEM3
G
NTRK2
N=2
N=0
AUH
TLE4
H
NTRK3
N=9
N=1
ETV6
EML4
AKAP13
RBPMS2
TSNARE1
I
RET
N=5
N=6
KIF5B
CCDC6
C10orf118
ERC1
J
ROS1
N=11
N=9
CD74
EZR
SLC34A2
TPM3
SDC4
Other

Supplementary Figure 4. CDx Gene Rearrangement Partners In DNA Versus RNA Proportion of RE partner genes detected in RNA versus DNA for A) *ALK*, B) *BRAF*, C) *FGFR2*, D) *FGFR3*, E) *NRG1*, F) *NTRK1*, G) *NTRK2*, H) *NTRK3*, I) *RET*, and J) *ROS1*. "Unknown" denotes partnerless RE. "Other" indicates partners only seen 1x in either DNA or RNA (but not in both). All partners are shown if there were <10 RE detected in that gene across all samples (DNA & RNA). Note this includes the dominant RNA and DNA events for all CDx RE+ samples (N = 148) regardless of whether they were detected in DNA, RNA, or both.

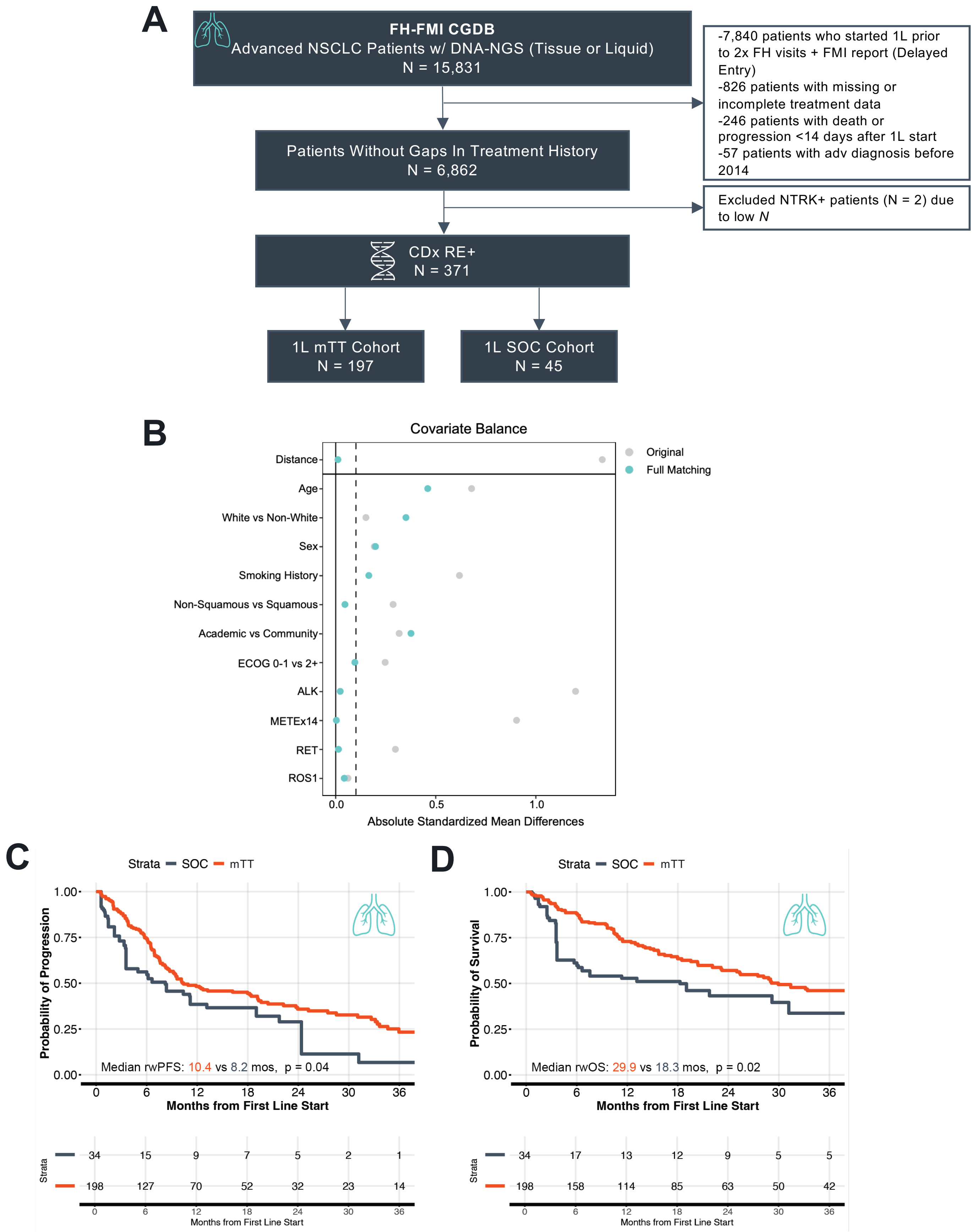

E

Survival	mTT % (95% CI)	SOC % (95% CI)
1-Year	73.0 (65.9-80.8)	52.8 (35.0-79.8)
2-Year	57.1 (49.4-66.1)	43.2 (25.9-72.2)

Supplementary Figure 5. Real-World Clinical Outcomes Of DNA-NGS Detected CDx RE+ Patients With NSCLC Receiving Matched Targeted Therapy (mTT) A) CONSORT diagram for advanced NSCLC CGDB outcomes analysis. B) Covariate balancing between the mTT and SOC cohorts using the Inverse Probability Of Treatment Weight (IPTW) method. Dashed line represents the absolute standardized mean difference (SMD) threshold of 10% which was used to assess balance. C) rwPFS, D) rwOS, and E) 1-year and 2-year survival of patients with CDx RE+ NSCLC receiving 1L mTT versus SOC. Data are from the Flatiron Health-Foundation Medicine Clinico-Genomic Database (FH-FMI CGDB). 1L, First Line; ICPI, Immune Checkpoint Inhibitor; mTT, Matched Targeted Therapy; NSCLC, Non-Small Cell Lung Cancer; SOC, Standard Of Care; RE, Rearrangement.

GENE	DOMAIN	DOMAIN EXONS	SPLICE VARIANTS	F1CDx COVERAGE
**ALK** NM_004304	TKD	20-29	.	All Exons; Intron 18, 19
**BRAF** NM_004333	TKD	11-18	.	All Exons; Intron 7-10
**FGFR2** NM_000141	TKD	11-17	.	All Exons; Intron 1, 17
**FGFR3** NM_000142	TKD	11-17	.	All Exons; Intron 17
**MET** NM_000245	TKD	15-21	METEx14 Ex1-13::Ex15-21	All Exons
**NRG1** NM_004495	EGF-Like	6	.	None*
**NTRK1** NM_002529	TKD	13-17	.	All Exons; Introns 8-11
**NTRK2** NM_006180	TKD	19-24	.	All Exons; Intron 12
**NTRK3** NM_002530	TKD	15-19	.	All Exons*
**RET** NM_020630	TKD	12-19	.	All Exons; Intron 7-11
**ROS1** NM_002944	TKD	36-42	.	All Exons; Intron 31-35

Supplementary Table 1. CDx Gene Rearrangement Active Domain/Splice Variant Definitions And F1CDx Coverage Asterisks (*) denote genes lacking coverage in hotspot intronic regions due to technical limitations of baiting in DNA. CDx, Companion Diagnostic; EGF-Like, Epidermal Growth Factor-Like ; F1CDx, FoundationOne CDx; TKD, Tyrosine Kinase Domain.

	DNA & RNA	DNA Only	RNA Only	Total Count
ALK	27	7	6	**40**
BRAF	10	7	10	**27**
METEx14	24	2	6	**32**
FGFR2	11	7	1	**19**
NTRK1/2/3	5	0	10	**15**
FGFR3	11	0	4	**15**
ROS1	7	2	4	**13**
NRG1	1	0	5	**6**
RET	5	1	0	**6**
All CDx Genes	101	26	46	**173**

Supplementary Table 2. CDx Gene Rearrangement Detection In Solid Tumors (N = 5129) Using Concurrent DNA-/RNA-NGS Count data underlying **Figure 1/Supplemental Figure 2**. N = 2 samples harbored multiple CDx gene RE (NSCLC w/ *ALK* [DNA & RNA] + *BRAF* [RNA Only]; CCA w/ *FGFR2* [DNA & RNA] + *BRAF* [RNA Only]). CDx, Companion Diagnostic.

	DNA & RNA	DNA Only	RNA Only	Total Count	ITT, N
ALK (NSCLC)	26	4	1	**31**	1305
BRAF (CNS)	1	2	2	**5**	44
METEx14 (NSCLC)	22	1	1	**24**	1305
NTRK1/2/3 (Solid Tumors)	5	0	10	**15**	5129
ROS1 (NSCLC)	7	1	3	**11**	1305
RET (Solid Tumors)	5	1	0	**6**	5129
FGFR2 (CCA)	4	0	0	**4**	93
NRG1 (NSCLC)	1	0	2	**3**	1305
FGFR3 (Bladder)	1	0	1	**2**	99
All CDx	72	9	20	**101**	5129

Supplementary Table 3. CDx Rearrangement Detection ITT Using Concurrent DNA-/RNA-NGS Count data underlying **Figure 2/Supplemental Figure 2/Supplemental Figure 3**. CDx, Companion Diagnostic; CCA, Cholangiocarcinoma; CNS, Central Nervous System; NSCLC, Non-Small Cell Lung Cancer; ITT, In Tumor Type.

Characteristic	Overall	CDx Gene RE(+)	CDx Gene RE(-)	p
N	1305	86	1219	-
Age At Bx (Years)				-
Median [IQR]	69 [63, 77]	69 [60, 75]	69 [63, 77]	0.35
<50 Years, N (%)	51 (3.9)	11 (12.8)	40 (3.3)	0.001*
Sex, N (%)				0.35
Female	646 (49.5)	48 (55.8)	598 (49.1)	-
Male	659 (50.5)	38 (44.2)	621 (50.9)	-
Genomic Ancestry†, N (%)				0.11
AFR	149 (11.4)	6 (7.0)	143 (11.7)	-
AMR	82 (6.3)	6 (7.0)	76 (6.2)	-
EAS	71 (5.4)	10 (11.6)	61 (5.0)	-
EUR	953 (73.0)	61 (70.9)	892 (73.2)	-
SAS	12 (0.9)	≤5 (2.3)	10 (0.8)	-
Unknown	38 (2.9)	≤5 (1.2)	37 (3.0)	-
Histology, N (%)				0.001*
Lung Adenocarcinoma	825 (63.2)	75 (87.2)	750 (61.5)	-
Lung Squamous Cell Carcinoma	305 (23.4)	6 (7.0)	299 (24.5)	-
NSCLC, NOS	149 (11.4)	4 (4.7)	145 (11.9)	-
Lung Large Cell Neuroendocrine Carcinoma	15 (1.1)	1 (1.2)	14 (1.1)	-
Lung Adenosquamous Carcinoma	4 (0.3)	0 (0.0)	4 (0.3)	-
Lung Large Cell Carcinoma	3 (0.2)	0 (0.0)	3 (0.2)	-
Lung Sarcomatoid Carcinoma	3 (0.2)	0 (0.0)	3 (0.2)	-
Lung Carcinosarcoma	1 (0.1)	0 (0.0)	1 (0.1)	-
Biopsy Site, N (%)				0.48
Primary	779 (59.7)	45 (52.3)	734 (60.2)	-
Lymph Node	221 (16.9)	19 (22.1)	202 (16.6)	-
Metastasis	196 (15.0)	15 (17.4)	181 (14.8)	-
Unknown	109 (8.4)	7 (8.1)	102 (8.4)	-
MSI Status				0.73
MSI-H	8 (0.6)	0 (0.0)	8 (0.7)	-
Not MSI-H	1103 (84.5)	71 (82.6)	1032 (84.7)	-
Unknown	194 (14.9)	15 (17.4)	179 (14.7)	-
TMB				<0.001*
≥10 Mut/Mb	353 (27.0)	6 (7.0)	347 (28.5)	-
<10 Mut/Mb	775 (59.4)	65 (75.6)	710 (58.2)	-
Unknown	177 (13.6)	15 (17.4)	162 (13.3)	-

Supplementary Table 4. Clinicogenomic Characteristics Of NSCLC Concurrent DNA-/RNA-NGS Cohort Stratified By CDx Rearrangement Status P values are multiplicity corrected and reflect comparison between the CDx RE(+) and (-) populations. Asterisks (*) indicate a significant P value (< 0.05). †Genomic Ancestry Reflects 1000 Genomes Project Super Populations: AFR=African; AMR=Admixed American; EAS=East Asian; EUR=European; SAS=South Asian. CDx, Companion Diagnostic; ITT, In Tumor Type; MSI-H, Microsatellite Instability-High; NOS, Not Otherwise Specified; NSCLC, Non-Small Cell Lung Cancer; RE, Rearrangement; TMB, Tumor Mutational Burden.

Characteristic	NTRK Fusion(+)	NTRK Fusion(-)	p
N	15	5114	-
Age At Bx (Years)			-
Median [IQR]	59 [29, 70]*	67 [60, 75]	0.12
<50 Years, N (%)	5 (33.3)	498 (9.7)	0.046*
Sex, N (%)			0.82
Female	8 (53.3)	2398 (46.9)	-
Male	7 (46.7)	2716 (53.1)	-
Genomic Ancestry†, N (%)			0.14
AFR	≤5 (26.7)	648 (12.7)	-
AMR	≤5 (26.7)	554 (10.8)	-
EAS	≤5 (6.7)	174 (3.4)	-
EUR	6 (40.0)	3535 (69.1)	-
SAS	0 (0.0)	55 (1.1)	-
Unknown	0 (0.0)	148 (2.9)	-
Biopsy Site, N (%)			0.82
Primary	9 (60.0)	2534 (49.6)	-
Lymph Node	0 (0.0)	486 (9.5)	-
Metastasis	4 (26.7)	1182 (23.1)	-
Unknown	2 (13.3)	912 (17.8)	-
MSI Status			0.50
MSI-H	1 (6.7)	105 (2.1)	-
Not MSI-H	13 (86.7)	4507 (88.1)	-
Unknown	1 (6.7)	502 (9.8)	-
TMB			0.82
≥10 Mut/Mb	1 (6.7)	748 (14.6)	-
<10 Mut/Mb	13 (86.7)	3841 (75.1)	-
Unknown	1 (6.7)	525 (10.3)	-
Cancer Types, N (%)			0.005*
Pancreatic	5 (33.3)	330 (6.5)	-
Sarcoma	2 (13.3)	268 (5.2)	-
CRC	1 (6.7)	618 (12.1)	-
Prostate	1 (6.7)	490 (9.6)	-
Breast	1 (6.7)	388 (7.6)	-
Head & Neck	1 (6.7)	143 (2.8)	-
Thyroid	1 (6.7)	47 (0.9)	-
CNS	1 (6.7)	43 (0.8)	-
Small Bowel	1 (6.7)	39 (0.8)	-
Benign	1 (6.7)	13 (0.3)	-

Supplementary Table 5. Clinicogenomic Characteristics Of Concurrent DNA-/RNA-NGS Solid Tumor Cohort Stratified By NTRK Fusion Status P values are multiplicity corrected and reflect comparison between the NTRK fusion(+) and (-) populations. Asterisks (*) indicate a significant P value (< 0.05). *Include N = 4 pediatric patients (≤18 years). †Genomic Ancestry Reflects 1000 Genomes Project Super Populations: AFR=African; AMR=Admixed American; EAS=East Asian; EUR=European; SAS=South Asian. CNS, Central Nervous System; CRC, Colorectal Cancer; MSI-H, Microsatellite Instability-High; TMB, Tumor Mutational Burden.

Characteristic	RET Fusion(+)	RET Fusion(-)	p
**N**	6	5123	-
**Age At Bx (Years)**			-
**Median [IQR]**	69 [69, 75]	67 [59, 75]	0.36
**<50 Years, N (%)**	0 (0.0)	503 (9.8)	>0.99
**Sex, N (%)**			>0.99
**Female**	3 (50.0)	2403 (46.9)	-
**Male**	3 (50.0)	2720 (53.1)	-
**Genomic Ancestry†, N (%)**			0.22
**AFR**	0 (0.0)	652 (12.7)	-
**AMR**	0 (0.0)	558 (10.9)	-
**EAS**	≤5 (33.3)	173 (3.4)	-
**EUR**	≤5 (66.7)	3537 (69.0)	-
**SAS**	0 (0.0)	55 (1.1)	-
**Unknown**	0 (0.0)	148 (2.9)	-
**Biopsy Site, N (%)**			>0.99
**Primary**	3 (50.0)	2540 (49.6)	-
**Lymph Node**	1 (16.7)	485 (9.5)	-
**Metastasis**	1 (16.7)	1185 (23.1)	-
**Unknown**	1 (16.7)	913 (17.8)	-
**MSI Status**			0.001*
**MSI-H**	0 (0.0)	106 (2.1)	-
**Not MSI-H**	1 (16.7)	4519 (88.2)	-
**Unknown**	5 (83.3)	498 (9.7)	-
**TMB**			0.001*
**≥10 Mut/Mb**	0 (0.0)	749 (14.6)	-
**<10 Mut/Mb**	1 (16.7)	3853 (75.2)	-
**Unknown**	5 (83.3)	521 (10.2)	-
**Cancer Types, N (%)**			>0.99
**NSCLC**	6 (100.0)	1299 (25.4)	

Supplementary Table 6. Clinicogenomic Characteristics Of Concurrent DNA-/RNA-NGS Solid Tumor Cohort Stratified By RET Fusion Status P values are multiplicity corrected and reflect comparison between the RET fusion(+) and (-) populations. Asterisks (*) indicate a significant P value (< 0.05). †Genomic Ancestry Reflects 1000 Genomes Project Super Populations: AFR=African; AMR=Admixed American; EAS=East Asian; EUR=European; SAS=South Asian. MSI-H, Microsatellite Instability-High; NSCLC, Non-Small Cell Lung Cancer; TMB, Tumor Mutational Burden.

Characteristic		mTT (N = 197)	SOC (N = 45)	p
**Sex**	M:F	38:62	47:53	0.41
**Median Age [IQR]**		67 [56, 75]	77 [69, 82]	<0.001*
**Race (%)**	White	128 (65.0)	26 (57.8)	0.44
	Black/African American	31 (15.7)	6 (13.3)	
	Other/Multiple Races	38 (19.3)	13 (28.9)	
**SES (%)**	1 - Lowest SES	25 (12.7)	4 (8.9)	0.69
	2	40 (20.3)	9 (20.0)	
	3	44 (22.3)	11 (24.4)	
	4	39 (19.8)	13 (28.9)	
	5 - Highest SES	49 (24.9)	8 (17.8)	
**Histology (%)**	Non-Squamous Cell Carcinoma	188 (95.4)	40 (88.9)	0.27
	Squamous Cell Carcinoma	9 (4.6)	5 (11.1)	
**Smoking Status (%)**	Hx Of Smoking	82 (41.6)	32 (71.1)	0.001*
	No Hx Of Smoking	115 (58.4)	13 (28.9)	
**Practice Type (%)**	Academic	33 (16.8)	2 (4.4)	0.08
	Community	164 (83.2)	43 (95.6)	
**ECOG (%)**	0	79 (40.1)	13 (28.9)	0.39
	1	87 (44.2)	21 (46.7)	
	2+	31 (15.7)	11 (24.4)	
**Biopsy Type (%)**	Tissue	160 (81.2)	40 (88.9)	0.40
	Liquid	37 (18.8)	5 (11.1)	
**CDx (%)**	*ALK*	118 (59.9)	5 (11.1)	<0.001*
	*MET*Ex14	43 (21.8)	29 (64.4)	
	RET	16 (8.1)	8 (17.8)	
	ROS1	20 (10.2)	3 (6.7)	
**Therapy Category (%)**	Chemo + ICPI	0 (0.0)	17 (37.8)	<0.001*
	ICPI	0 (0.0)	28 (62.2)	
	mTT	197 (100.0)	0 (0.0)	

Supplementary Table 7. Clinical Characteristics Of Patients With Advanced NSCLC And CDx Rearrangements In A Real-World Clinical Cohort (FH-FMI CGDB) Asterisks (*) indicate a significant P value (< 0.05). Data are from the Flatiron Health-Foundation Medicine Clinico-Genomic Database (FH-FMI CGDB). CDx, Companion Diagnostic; ICPI, Immune Checkpoint Inhibitor; mTT, Matched Targeted Therapy; NSCLC, Non-Small Cell Lung Cancer; SES, Socioeconomic Status; SOC, Standard Of Care.

SUPPLEMENTARY MATERIALS & METHODS

Real-World Clinical Outcomes In NSCLC With CDx Rearrangements (RE) Receiving Matched Targeted Therapy

Clinicogenomic Cohort Selection

NCSLC clinical outcomes were assessed using the nationwide U.S.-based Flatiron Health-Foundation Medicine Clinico-Genomic Database (FH-FMI CGDB). Retrospective longitudinal clinical data were derived from electronic health records (EHR), comprising patient-level structured and unstructured data curated via technology-enabled abstraction of clinical notes and radiology/pathology reports, from the Flatiron Health Research Database[1] and linked to genomic data derived from FMI testing (FoundationOne®, F1CDx, or FoundationOne®Liquid CDx [F1LCDx®]) by deidentified, deterministic matching[2]. Clinical data originated from approximately 280 US cancer clinics (~800 sites of care). This study included 6,862 patients with advanced NSCLC diagnosed between January 2014 and September 2024. The study population included patients with detected CDx RE: *ALK* fusions or rearrangements; *MET*Ex14 splice variants; or *NTRK*, *RET*, or *ROS1* fusions. Real-world progression-free survival (rwPFS) and real-world overall survival (rwOS) on first-line (1L) therapy were compared between CDx-positive patients who received a mTT versus patients who received SOC therapies. mTT included drugs associated with a tissue sequencing-based CDx (**Supplementary Table 8**). The SOC treatment cohort included patients who received 1L chemotherapy plus an immune checkpoint inhibitor (ICPI) or ICPI monotherapy and excluded patients who 1) ever received a clinical study drug or 2) received a mTT in any line other than 1L. Approval of the study protocol was obtained from the WCG Institutional Review Board prior to study conduct and included a waiver of informed consent based on the observational, noninterventional nature of the study.

Statistical Analysis – Clinicogenomic Cohort

rwPFS was calculated from the start of 1L treatment to disease progression or death and patients with no record of progression or death were right-censored at the last clinic note date. rwOS was calculated from the start of 1L treatment to death and patients with no record of mortality were right-censored at the last date of confirmed activity. To account for immortal time, patients with delayed entry who started 1L treatment prior to two Flatiron Health site visits and receipt of an FMI testing report were excluded from the analysis cohort. Due to the presence of non-proportional hazards, differences in rwOS and rwPFS were assessed with a weighted log-rank test with weights from the Fleming & Harrington rho-gamma family (ρ=1, γ=1) to account for the delayed treatment effects

observed in the ICPI-based SOC regimens[3]. Inverse probability of treatment weighting (IPTW) was used to address potential confounders due to clinical imbalances between treatment cohorts. Missing values were imputed with the expected values based on observed covariates using random forests (R package 'missForest'). Covariates included in the propensity model were: age, race, sex, smoking history, histology, practice type, ECOG PS, and CDx gene. Standardized mean difference (SMD) was used to assess balance and +/-10% was considered acceptable. Age, race, practice type, smoking status, and sex remained unbalanced with an absolute SMD ≥10% after matching.

SUPPLEMENTARY REFERENCES

1 Flatiron Health Database Characterization Guide. 2025. Available at https://flatiron.com/database-characterization. Accessed April 18, 2025.

2 Singal G, Miller PG, Agarwala V et al. Association of Patient Characteristics and Tumor Genomics With Clinical Outcomes Among Patients With Non–Small Cell Lung Cancer Using a Clinicogenomic Database. JAMA 2019;321:1391–1399.

3 Klinglmüller F, Fellinger T, König F et al. A Comparison of Statistical Methods for Time-To-Event Analyses in Randomized Controlled Trials Under Non-Proportional Hazards. Stat Med 2025;44:e70019.

CDx Gene	CDx mTT	Non-CDx mTT
ALK	Alectinib	Lorlatinib
	Brigatinib	Ensartinib
	Ceritinib	
	Crizotinib	
MET	Capmatinib	Cabozantinib
		Crizotinib
		Savolitinib
		Tepotinib
NTRK	Entrectinib	Repotrectinib
	Larotrectinib	
RET	Pralsetinib	
	Selpercatinib	
ROS1	Crizotinib	Repotrectinib
	Entrectinib	

Supplementary Table 8. Flatiron Health-Foundation Medicine CGDB Cohort Targeted Therapy Inclusion Criteria mTT associated with each CDx rearrangement gene included in the NSCLC outcomes analysis cohort. CDx mTT include those with a defined tissue DNA-NGS-based CDx. The 1L mTT analysis cohort included CDx driver-positive patients treated with a corresponding CDx mTT. Patients who received either a CDx mTT or a non-CDx mTT agent at any other time (i.e., non-1L) were excluded from the SOC analysis cohort. CGDB, Clinico-Genomic Database; mTT, Matched Targeted Therapy; SOC, Standard Of Care.
